# Spatiotemporal connectivity dynamics in spatially structured populations

**DOI:** 10.1111/1365-2656.13783

**Published:** 2022-07-30

**Authors:** Joseph Drake, Xavier Lambin, Chris Sutherland

**Affiliations:** ^1^ Department of Environmental Conservation University of Massachusetts‐Amherst Amherst MA USA; ^2^ Organismal and Evolutionary Biology Interdisciplinary Program University of Massachusetts‐Amherst Amherst MA USA; ^3^ School of Biological Sciences University or Aberdeen Aberdeen UK; ^4^ Centre for Research into Ecological and Environmental Modelling University of St Andrews St Andrews UK

**Keywords:** Bayesian, colonization–extinction, mammal, population dynamics, spatially realistic metapopulation model, SPOM, stochastic patch occupancy model, structural connectivity

## Abstract

1. Connectivity is a fundamental concept linking dispersal to the emergent dynamics and persistence of spatially structured populations. Functional measures of connectivity typically seek to integrate aspects of landscape structure and animal movement to describe ecologically meaningful connectedness at the landscape and population scale.
2. Despite this focus on function, traditional measures of landscape connectivity assume it is a static property of the landscape, hence abstracting out the underlying spatiotemporal population dynamics. Connectivity is, arguably, a dynamic property of landscapes, and is inherently related to the spatial distribution of individuals and populations across the landscape. Static representations of connectivity potentially overlook this variation and therefore adopting a dynamic approach should offer improved insights about connectivity and associated ecological processes.
3. Using a large‐scale, long‐term time series of occupancy data from a metapopulation of water voles *Arvicola amphibius*, we tested competing hypotheses about how considering the dynamic nature of connectivity improves the ability of spatially explicit occupancy models to recover population dynamics. Iteratively relaxing standing assumptions of connectivity metrics, these models ranged from spatially and temporally fixed connectivity metrics that are widely applied, to the more flexible, but lesser used model that allowed temporally varying connectivity measures that incorporate spatiotemporally dynamic patch occupancy states.
4. Our results provide empirical evidence that demographic weighting using patch occupancy dynamics and temporal variability in connectivity measures are important for describing metapopulation dynamics.
5. We highlight the implications of commonly held assumption in connectivity modelling and demonstrate how they result in different and highly variable predictions of metapopulation capacity. Thus, we argue that the concept of connectivity and its potential applications would benefit from recognizing inherent spatiotemporal variation in connectivity that is explicitly linked to underlying ecological state variables.

## Graphical abstract

Spatiotemporal connectivity depends on both the landscape network and the distribution of connected populations. The authors demonstrate the dynamic nature of connectivity and show its contributions to population processes using a bespoke Bayesian stochastic patch occupancy model that accounts for temporally varying dispersal and the spatial distribution of dispensers.

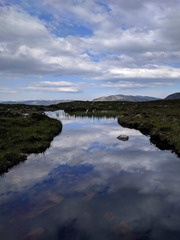

## 1 INTRODUCTION

Dispersal is a key, but complex, ecological process that impacts local population dynamics and, through resulting connectivity, shapes the emergent dynamics and ultimate persistence of spatially structured populations (Bowler & Benton, 2005; Clobert et al., 2009; Drake et al., 2021). Dispersal is generally defined as the movement between natal and breeding patches (Clobert et al., 2012; Matthysen, 2012), and connectivity is the aggregate strength of these linkages among habitat patches (Calabrese & Fagan, 2004). As such, connectivity represents the set of spatial dependencies that arise between individuals in a landscape (Kool et al., 2013) and offers a lens through which to view a suite of complex processes, which themselves are challenging to observe directly (Clobert et al., 2009).

Connectivity lies squarely at the centre of contemporary conservation science (Elliot et al., 2014), yet approaches to quantifying connectivity often lack the mechanistic basis required to make them informative of realized connectivity on the landscape. To date, connectivity has generally been treated as a static feature of a system (Kool et al., 2013; but see Fernández et al., 2016) and there have been calls for a greater appreciation for dynamic nature of connectivity (e.g. McIntyre et al., 2018) and a focus on long‐term changes in habitat due to environmental change (Bishop‐Taylor et al., 2018) or climate change (Drake et al., 2017; Ruiz et al., 2014). Indeed, modification of connectivity through short‐term changes in habitat (Martensen et al., 2017) or loss of connections between patches (Perry & Lee, 2019) have been shown to have implications for metapopulation functioning. So, while a focus on the shifting landscape mosaic is important when considering potential connectivity, the contribution of dispersers and their spatial distribution may be equally or more important to consider; this has often been overlooked even despite the implicit focus on movement vital for realized connectivity (Drake et al., 2021). This raises questions about the utility of inferred connectivity, particularly for future projections, and especially for non‐equilibrium populations (Johansson et al., 2013). Also, many approaches for quantifying connectivity typically ignore the underlying spatial distribution, and heterogeneity therein, of the dispersing individuals, thus assuming spatially and temporally homogeneous contribution to connectivity across studied systems (Zeller et al., 2020).

Spatially realistic metapopulation theory (Hanski, 1999; Hanski & Ovaskainen, 2003) is one framework in which the dynamic nature of connectivity is made explicit: connectivity is treated as a landscape aggregate of weighted patch contributions, where the weighting scheme relates directly to the occupancy state of a patch (i.e. are dispersers present?), the size of the population occupying the patch (i.e. how many potential dispersers are present?) and the dispersal behaviour (i.e. how far will dispersers travel and what controls dispersal?) all of which may change in space and time. Weighting connectivity estimates on any number of ecological state variables or demographic data should be increasingly possible with the proliferation of species distribution models (Acevedo et al., 2017; Ovaskainen et al., 2016), occupancy models (MacKenzie et al., 2018) and abundance models (Kery & Royle, 2016) that offer frameworks for spatially explicit predictions of ecological state variables at landscape scales. Therefore, there is no reason weighting schemes applied in metapopulation models cannot be formally integrated into connectivity research in general (Meyer et al., 2020; Morin et al., 2017; Sutherland et al., 2014). Metapopulations represent ideal systems in which to investigate the consequences of the restrictive assumptions of spatiotemporal invariance for inference about connectivity, a topic that has hitherto received little attention (Perry & Lee, 2019), despite its potential to fundamentally alter predictions about landscape connectivity.

Acknowledging that the definition of connectivity is tied closely to a specific scale, context and available data, the existence of a single unifying measure is unlikely. Instead, we strive to better understand how specific assumptions impact model outcomes and inference so they can be applied sensibly and judiciously. We address this through the analysis of data collected from a long‐term, large‐scale model mammalian metapopulation to evaluate how predictions of metapopulation dynamics and persistence are influenced by commonly held assumptions of connectivity. Bayesian analysis of stochastic patch occupancy models (SPOMs: Ovaskainen, 2002, Ovaskainen & Hanski, 2004), a flexible class of metapopulation models, lends itself naturally to the relaxation of the implicit assumptions often made in landscape ecology about spatiotemporal (in)variability of model parameters and patch occupancy states, and to formal comparison of model performance. We analyse a patch occupancy time series using a spatiotemporally homogeneous metapopulation model, that is, one that assumes all patches are occupied and that dispersal parameters are temporally invariant. We then iteratively relax the assumptions of spatial and temporal invariance in connectivity; this is analogous to increased realism in how demographic contributions to connectivity are characterized. Relaxing these rigid spatiotemporal connectivity model assumptions should provide greater insight to sources of variation in the dispersal process which drive occupancy patterns and colonization–extinction processes. Our approach seeks to quantify the relative contributions of spatial and temporal variability in demographic contributions to connectivity dynamics, and in doing so attempts to advance the ideas of demographic connectivity. While we demonstrate this using a metapopulation ecological modelling framework, we believe the concept could be applied to ecological connectivity‐related research in general.

## 2 MATERIALS AND METHODS

### 2.1 Study system

We focus on a model mammalian metapopulation system in Assynt, northwest Scotland, UK. The species is the riparian specialist water vole *Arvicola amphibius*, and the patch network is a riparian network consisting of 98 vegetated patches embedded in an approximately 140 km^2^ area. Around 10% of the total 860 km waterway network represents suitable habitat, patches are therefore highly fragmented (mean nearest neighbour distance of 0.5 km) and vary in size from 50 m to nearly 3 km (mean = 0.847 km). The patches represent temporally stable habitat with very little variation observed over 20 years and/or between periods of occupancy (Bryce et al., 2013). The intervening landscape is almost exclusively composed of unsuitable heather matrix through which water voles disperse overland and within the riparian network (Drake, 2021; Fisher et al., 2009; Telfer et al., 2003). Patches are connected by dispersal, they exhibit frequent turnover, and the metapopulation fluctuates around a long‐term average of 55% occupancy, that is, the system functions as a classic metapopulation (Sutherland, 2013; Sutherland et al., 2012). Between 1999 and 2015, the water vole patches were surveyed between 1 and 4 times per year during the breeding season (July and August). Surveys involved faecal latrine searches as indicators of vole occupancy. The data are year‐ and patch‐specific binary detection histories representing imperfect observations of patch occupancy for a diffuse patch network that lends itself naturally to analysis using spatial occupancy models (see below). This study complied with all pertinent local and national legislation and regulations for the duration of the study; no animals were handled for the data used in this study and ethical approval for this research was not needed. For further details on the study system and data collections, see Sutherland et al. (2012, 2013, 2014).

### 2.2 Spatial occupancy modelling framework

The 17‐year 98‐patch time series of detection/non‐detection data was analysed using a Bayesian spatial occupancy model (Chandler et al., 2015; Risk et al., 2011; Sutherland et al., 2014). Here, the latent patch occupancy state, z, is treated as a partially observed Bernoulli random variable, with site (*i*) and year (*t*) specific occupancy probability $\psi_{i,t}$. In the initial year, where no information about occupancy states or dynamics in the previous year are available, occupancy is modelled as:

(1) $$z_{i,1}∼\text{Bernoulli}\left(\psi_{1}\right),$$

where $\psi_{1}$ is the expected proportion of occupied sites in the initial year (1999). In subsequent years (i.e. *t* > 1), occupancy states are modelled as:

(2) $$z_{i,t}∼\text{Bernoulli}\left(\psi_{i,t}\right),$$

where occupancy probability is a Markovian process that depends on the occupancy state in the previous year and conditional colonization ($\gamma_{i,t}$, if $z_{i,t-1}$ = 0) and extinction ($\varepsilon_{i,t}$, if $z_{i,t-1}$ = 1) probabilities:

(3) $$\psi_{i,t}=\left(1-z_{i,t-1}\right)\gamma_{i,t}+z_{i,t-1}\left(1-\varepsilon_{i,t-1}\right).$$

Assuming that patch size and population size are correlated (Sutherland et al., 2014), the probability of extinction, $\varepsilon_{i,t}$, is modelled as a function of patch size, here the length of the riparian habitat patch, using a logit linear model:

(4) $$\text{logit}\left(\varepsilon_{i,t}\right)=\delta_{0}+\delta_{1}A_{i},$$

where $A_{i}$ is the time invariant length of a patch *i* and $\delta_{0}$ and $\delta_{1}$ are the regression parameters to be estimated.

Unoccupied sites are assumed to be (re)colonized with probability $\gamma_{i,t}$, which is modelled as an asymptotically increasing function of connectivity ($S_{i,t}$):

(5) $$\gamma_{i,t}=1-\exp\left(-S_{i,t}\right),$$

The general formulation of the connectivity term, which we adapt below, is given by:

(6) $$S_{\mathrm{it}}^{*}=\beta \sum_{j\neq i}A_{i}z_{i,t}\exp\left(-\alpha d_{i,j}\right),$$

where β is the per capita effective dispersal rate parameter, $A_{i}$ is the patch length and $z_{i,t}$ is the patch state which sets the contributions of empty patches to zero. The term $\exp\left(-\alpha d_{i,j}\right)$ is a spatial function that declines with inter‐patch distance, $d_{i,j}$, the spatial scale of the decline being determined by the scale parameter α. This function can be thought of as a dispersal kernel and is the spatial weighting that defines the distance‐dependent contribution of a patch to the connectivity of all other patches.

To evaluate specific assumptions influence on estimates of model parameters, and the corresponding inference to connectivity, we define four alternative formulations of Equation 6. These formulations are focused on two aspects of the model and data that broadly represent analogies of commonly made assumptions in connectivity modelling. The first relates to the *structural connectivity* paradigm that defines connectivity as a property of the landscape rather than the populations residing within them (Urban & Keitt, 2001), and the second relates the definition of ‘function’ in the *functional connectivity* paradigm which seeks to introduce aspects of species movement ecology (Adriaensen et al., 2003). This iterative relaxation of connectivity modelling assumptions reflects increasing realism of connectivity estimated from occupancy data with focus on demographic contributions and helps account for shifts in dispersers (Drake et al., 2021). We refer to these as *unweighted with time‐invariant dispersal* (UI), *demographically weighted with time‐invariant dispersal* (DI), *unweighted with time‐varying dispersal* (UV) and *demographically weighted with time‐varying dispersal* (DV). Full connectivity model formulations and descriptions are provided in Table 1.

**TABLE 1:** Alternative formulations of the standard metapopulation connectivity function. Connectivity (S) is modelled as a function of patch size ($A_{i}$), a proxy for population size and a distance‐dependent spatial function $e^{\left(-\alpha d_{i,j}\right)}$. The occupancy column relates to the structural assumpion about contributions to connectivity and the dispersal column relates to functional assumptions about the temporal nature of dispersal. The Gibbs variable selection (GVS) column is a summary of the model support based on the posterior distribution of the indicator variable used in the GVS

Definition	Occupancy	Dispersal^a^	Equation	GVS
*Unweighted with time‐invariant dispersal* (UI)	$z_{i,t}\equiv 1$	α and β	$S_{i,t}^{\mathrm{UI}}=\beta \sum_{j\neq i}A_{i}e^{\left(-\alpha d_{i,j}\right)}$	0.0011
*Unweighted with time‐varying dispersal* (UV)	$z_{i,t}\equiv 1$	$\alpha_{t}$ and $\beta_{t}$	$S_{i,t}^{\mathrm{UV}}=\beta_{t}\sum_{j\neq i}A_{i}e^{\left(-\alpha_{t}d_{i,j}\right)}$	0.0078
*Demographically weighted and time‐invariant dispersal* (DI)	$z_{i,t}$	α and β	$S_{\mathrm{it}}^{\mathrm{DI}}=\beta \sum_{j\neq i}A_{i}e^{\left(-\alpha d_{i,j}\right)}z_{i}$	0.1543
*Demographically weighted and time‐varying dispersal* (DV)	$z_{i,t}$	$\alpha_{t}$ and $\beta_{t}$	$S_{\mathrm{it}}^{\mathrm{DV}}=\beta_{t}\sum_{j\neq i}A_{i}e^{\left(-\alpha_{t}d_{i,j}\right)}z_{i}$	0.8368

^a^ For models without temporally varying connectivity, the parameters β and α are static, whereas in models with temporally varying connectivity it is treated as a year‐specific random effect where $\theta_{t}=\theta +\epsilon_{t}$, where $\theta_{t}\sim \text{Normal}\left(0,\sigma_{\theta }^{2}\right)$ and $\theta =\left\{\alpha ,\beta \right\}$.

We represent the structural assumption (models UI and UV) by setting all patches in the network to be occupied ($z_{i,t}=z=1$) which produces a measure of connectivity that is the aggregate of spatiotemporally homogeneous contributions from every patch weighted by their size which is heterogeneous in space but temporally invariant. We then relax that assumption by adopting the classical metapopulation formulation of the model where patch contributions are weighted also by the occupancy state, which is spatiotemporally dynamic (models DI and DV). We account for the functional assumption through inclusion of the dispersal function that is also typically assumed to be temporally invariant (but see Andrew & Ustin, 2010). Here, contributions to connectivity are defined by the per‐capita effective dispersal rate (β) and the scale parameter (α). By setting these parameters as $\alpha_{t}=\alpha$ and $\beta_{t}=\beta$, respectively, we enforce temporal invariance (models UI & DI). We then relax that assumption (models UV and DV) to allow for dynamic dispersal estimates by modelling year specific dispersal parameters (i.e. $\alpha_{t}$ and $\beta_{t}$) as random deviates coming from a hyper distribution: $\theta_{t}=\theta +\epsilon_{t}$, where $\theta_{t}\sim \text{Normal}\left(0,\sigma_{\theta }^{2}\right)$ and $\theta =\left\{\alpha ,\beta \right\}$.

Finally, acknowledging that the data, $y_{i,j,t}$, denoting whether latrines were detected during the *j*th visit to patch *i* in year *t*, arise via an imperfect observation process, we assume the data are conditional on the estimated latent occupancy state z:

(7) $$y_{i,j,t}\;|\;z_{i,t}∼\text{Bernoulli}\left(z_{i,t}p_{t}\right),$$

treating year‐specific detection probabilities as random effects (Sutherland et al., 2014):

(8) $$\text{logit}\left(p_{t}\right)∼\text{Normal}\left(\mu_{p},\sigma_{p}\right),$$

### 2.3 Model comparison

We use a Gibbs variable selection (GVS) approach (O'Hara & Sillanpaa, 2009; Tenan et al., 2014) to quantify support for competing model structures. In the GVS approach, the indicator variables represent variables that define specific model structures, and the mass of the posterior distribution of the indicator variables corresponds to support for hypothesized connectivity model formulations. We introduce two latent indicator variables, $I^{z}$ and $I^{D}$ that correspond to the weighting scheme (i.e. whether occupancy state weighting is included or not), and the random effect structure of the dispersal parameters, $\alpha_{t}$ and $\beta_{t}$, respectively. These enter the model as:

(9) Sit=∑Ai×exp−αdij,ifIz=0∑Ai×zi×exp−αdij,ifIz=1,

for the occupancy weighting, and as $\beta_{t}=\beta +\epsilon_{\beta ,t}\times I^{D}$ and $\alpha_{t}=\alpha +\epsilon_{\alpha ,t}\times I^{D}$, which imposes temporal invariance on effective dispersal when $I^{D}=0$. For both binary indicator variables, we used a $I∼\text{Bernoulli}\left(0.5\right)$ prior, assuming no prior information about support for either outcome, and hence posterior distributions that deviate from 0.5 suggest support or not for specific assumptions. This offers a means by which to assess statistical support for the four hypothesized forms of connectivity.

However, we were also interested in evaluating the ecological significance of connectivity assumptions and do so by calculating the metapopulation capacity (MC) under each of the models. MC incorporates measures of patch area, connectivity, spatial network structure and dispersal behaviour to quantify the relative ability to support metapopulations in a spatially explicit metric (Hanski & Ovaskainen, 2000; Schnell et al., 2013). While generally used in analyses comparing scenarios of augmented networks, here, for a single network but with competing models, it can be instructive to evaluate how sensitive this important measure is to specific connectivity modelling assumptions. Therefore, by holding all other aspects of MC static, we can explore the impact of competing estimates of dispersal parameters among our models to see the impact to MC and the implied long‐term persistence of patch networks through time.

Each of the models were analysed using Markov chain Monte Carlo, fitted in R 3.6.1 (R Core Team, 2019) using the r package nimble (de Valpine et al., 2017), with three chains of 100,000 iterations, 50,000 discarded for burn‐in. Model priors (see Appendix S1) were chosen to be non‐informative (Gelman, 2006; Gelman et al., 2017). Prior sensitivity analysis, based on visual inspection of posteriors, suggested that inference was not sensitive to prior specification. Visual diagnostics of model chains as well as autocorrelation lag plots and *r‐hat* values provided evidence of convergence (Plummer et al., 2006). Using parameter estimates from each connectivity model, we used the r package metacapa (Strimas‐Mackey & Brodie, 2018a) to calculate metapopulation capacities using the joint posterior distribution of parameters from the metapopulation model; thus, we are able to report point estimates of MC with associated uncertainty. All visualizations were produced using the r package ggplot2 (Wickham, 2016). Parameter estimates are presented as posterior means, unless otherwise noted, with 95% credible intervals (CI's).

## 3 RESULTS

We found substantial support for the demographically weighted with time‐varying dispersal hypothesis (DV: $\mathrm{pr}\left(I^{Z}+I^{D}=2\right)=0.84$, Table 1). Considering GVS‐based support for each hypothesis was calculated separately, in relative terms, models containing demographic weighting carried slightly more combined model weight ($\mathrm{pr}\left(I^{z}=1\right)=0.99$ and $\mathrm{pr}\left(I^{D}=1\right)=0.85$, Table 1). Also, support for uninformed and invariant model was negligible (UI: $\mathrm{pr}\left(I^{Z}+I^{D}=0\right)$ < 0.01, Table 1). Thus, we provide evidence of dynamic connectivity in spatially structured populations and the importance of considering spatiotemporal weighting related to both the underlying state‐variable and the strength and scale of connectivity.

**FIGURE 1:**
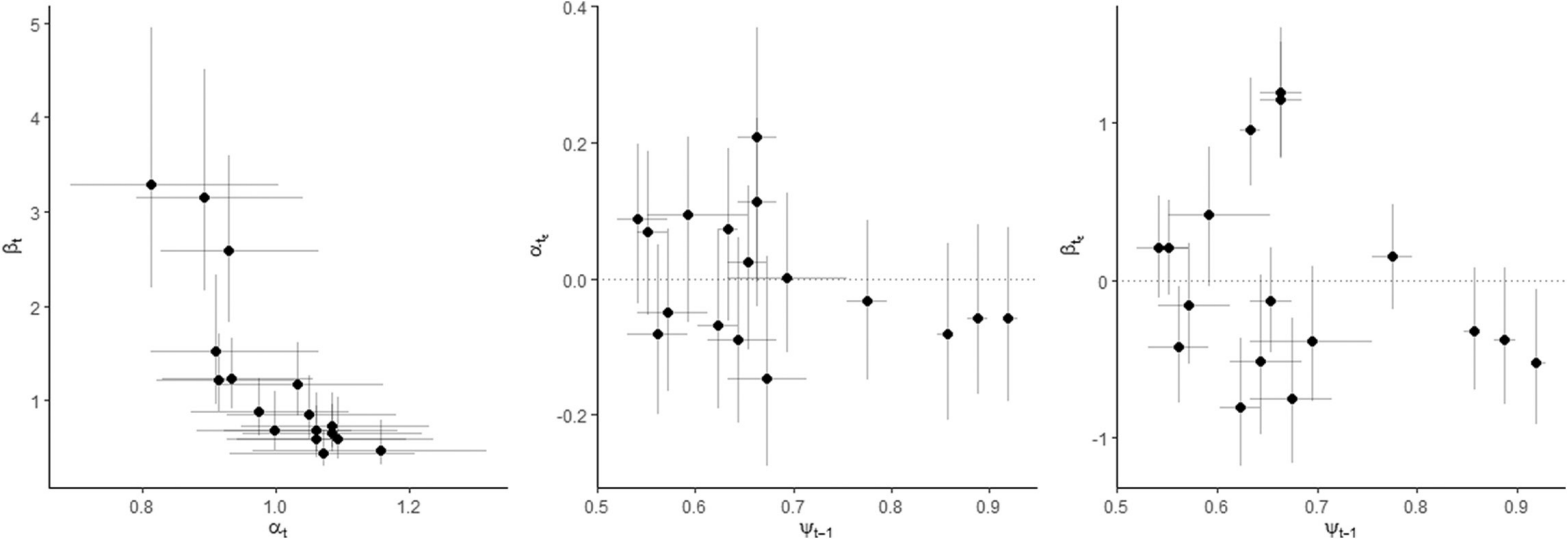
The random effect variance relationship of raw parameter estimates representing our connectivity process from the demographically weighted, time‐varying model (DV) to each other and estimated occupancy in the previous years. This represents the underlying raw parameter estimates variance around the mean of the random effect. Left column: The random effect variances, $\epsilon_{t}$, for the rate of effective dispersal $\beta_{t}$ and dispersal scaling parameters $\alpha_{t}$. Middle column: Random effects variance of $\alpha_{t}$ and previous years occupancy estimates, $\psi_{t-1}$. Right column: Random effects variance of $\beta_{t}$ and previous years occupancy estimates, $\psi_{t-1}$. Error bars represent 50% credible interval (CI) for parameter values.

The support for the inclusion of the occupancy weighting (models DI and DV) is compelling and intuitive: connectivity measured as a function of occupied patches (rather than all) better predicts occupancy dynamics as it includes information about the spatial distribution of potential dispersers. The support for temporal variability (models UV and DV) in the strength of connectivity is interesting (Figure 1) and deserves further discussion (see below). The dispersal kernel is defined by the scaling parameter, α, and the rate of effective dispersal, β. For the time invariant models UI and DI, α was 0.461 [0.171–0.881] and 0.446 [0.236–0.0.738] for the unweighted and weighted models, respectively (Appendix S2). Estimates of the average α (i.e. the mean of the random effect distribution) for the models UV & DV which allowed temporal variability via a random effect were 0.518 [0.210–1.04] and 0.588 [0.316–1.084], for UV and DV, respectively. Year‐specific estimates ranged from 0.463 [0.113–0.946] to 0.729 [0.212–2.022] for UV and 0.513 [0.205–0.94] to 0.780 [0.289–2.189] for the DV (Appendix S2). Thus, estimates of the scale of dispersal are higher without weighting, and although average values are similar between temporally varying and invariant models, there exists substantial interannual variation. For β, estimates from the static models were 0.096 [0.029–0.229] and 0.128 [0.056–0.259] for UI and DI, respectively, compared to corresponding random effects average estimates of 0.087 [0.027–0.242] and 0.144 [0.054–0.388] for the time‐varying models UV and DV, respectively. Yearly estimate of β ranged from 0.058 [0.010–0.190] to 0.313 [0.059–0.924] for UV and 0.085 [0.014–0.268] to 0.588 [0.132–1.915] for DV (Appendix S2). For dispersal rate, estimates are lower without weighting, and again, while average values are similar between temporally varying and invariant models, there exists substantial interannual variation. In general, the inclusion of either demographic weighting or temporally varying dispersal parameters (i.e. increased realism) produces shorter dispersal distances (1/α) and higher per capita dispersal rates (β; Appendix S2).The temporally dynamic model parameters $\alpha_{t}$ and $\beta_{t}$, the width and height of the kernel, respectively, were negatively correlated (Figure 1a), and interestingly, the observed temporal variability in both $\alpha_{t}$ and $\beta_{t}$ were not related to annual metapopulation size (number of occupied patches) in an obvious way (Figures 1b,c). Differences among patch occupancy estimates were negligible among models (Appendix S2).

**FIGURE 2:**
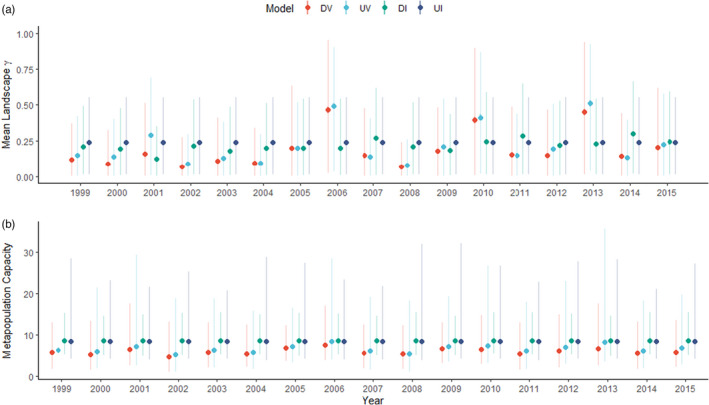
Descriptions of local and landscape level processes may depend on the model of connectivity used and its underlying assumptions such as if they are demographically weighted and time‐varying (DV), unweighted and time‐varying (UV), demographically weighted and time‐invariant (DI) or unweighted and time‐invariant (UI). (2a) Annual measures of total colonization probability under each of the four connectivity parameterizations of the stochastic patch occupancy model (SPOM). The measures show the landscape‐level summary of individual landscape pixel colonization probabilities. Points represent the average across all pixels. The vertical lines represent the 95% interval spanning the 0.025 and 0.975 quantiles of the empirical distribution of landscape colonization probabilities, product of the realized connectivity between patches (see Appendix S3). (2b) Annual metapopulation capacity (MC; Hanski & Ovaskainen, 2000) calculated using the joint posterior distribution of parameter estimates for each of the four connectivity parameterizations of the SPOM. Points represent the posterior means MC and vertical lines are the 95% Bayesian credible intervals (CIs). These CIs provide insight into the level of noise surrounding the point estimates of metapopulation capacities which are almost exclusively ignored in the literature.

To understand how estimates of connectivity model parameters translate to characterizations of landscape connectivity, we calculated 2‐year‐specific measures of total connectivity for each of the four formulations. We used the model‐specific connectivity functions and naïve occupancy values, for convenience and relative comparisons across models. First, we computed the *landscape‐level* average colonization probability (i.e. from Equation 5) which is the average colonization probability across each pixel of a raster defined as a rectangular polygon contained within a 2 km buffer around the patches in the network (Appendix S3). Second, we calculated a *network‐level* average colonization probability which is the average colonization probability across each patch in the network. Average landscape‐level colonization was lowest for the dynamic models (DV = 0.187 [0.175–0.201]; UV 0.214 [0.201–0.227], Figure 2a), while the static, unweighted model had the highest 17‐year mean (UI = 0.240 [0.226–0.253], Figure 2a). Comparing the range of annual values, however, changes this trend with the dynamic, weighted model having the largest range of annual colonization means (DV range = 0.067 [0.061–0.074] to 0.464 [0.441–0.489], Figure 2a). However, inclusion of only a demographic weighting allowed a temporal realism to emerge, but with a smaller range of values (DI: 17 year mean = 0.218 [0.205–0.231]; range = 0.125 [0.116–0.134] to 0.301 [0.285–0.317]). This is likely a result of underlying spatiotemporally heterogeneity in demographic covariate weighting influencing the static, weighted model results. Network‐level colonization estimates followed similar trends (see Appendix S3). In general, including demographic weighting, temporally varying dispersal kernels, or both (i.e. increased realism) induces heterogeneity in the realized functional landscape connectivity, while static and invariant measures estimate greater connectivity on average. We also conducted a Freeman–Tukey Goodness‐of‐Fit test (sensu Kery & Royle, 2021) which did not produce evidence of a lack of fit (Appendix S4).

MC, the measure of relative potential of a landscape to maintain persisting metapopulations, was lowest when connectivity was assumed to be a fully dynamic property of the system regardless of the weighting structure used (Figure 2b). In contrast, the weighting scheme for the (less supported) temporally invariant models did affect predictions of MC: assuming all patches are occupied results in a less precise estimates of MC when compared to estimates from the demographically weighted connectivity model (UI MC = 8.41 [4.16–25.78] and DI MC = 8.66 [5.18–15.08], respectively). Estimated MC, for most supported model including demographic weighting with temporally varying dispersal, was less than the static‐structural model (DV: mean capacity = 6.03, range = 4.84 [1.10–13.14] to 7.58 [3.95–17.02]). For both dynamic models, MC was highest in 2006 (Figure 2b), still lower than static metrics (Figure 2b).

Here we produce seldomly reported quantification of the uncertainty associated with estimates of MC, and the first that we are aware of with full joint posterior distributions of model parameters. Interestingly, the homogeneous model that produces estimates of temporal averages of time‐varying parameters and makes unrealistic assumptions about the distribution of potential dispersers has extremely large degree of uncertainty which may render them useless from an applied perspective (Figure 2b). Increases in biological realism reduce uncertainty in resulting estimates of MC, although predictions appear more sensitive to the use of estimates from models that allow for temporal invariance than use realistic representations of the distribution of potential dispersers (Figure 2b).

## 4 DISCUSSION

We present an empirical evaluation of two widespread assumptions used in the generation of connectivity metrics. In an attempt to understand how characterizations of connectivity propagate through characterizing the dynamics of spatially structured populations, we advance discussions about the dynamic nature of connectivity. We show that spatiotemporal assumptions about effective dispersal rates and the underlying distributions of the potential pool of dispersers influence statistical estimation and ecological inference using spatially explicit SPOMs. We add empirical weight to the theoretical assertion that it *is* important to consider connectivity dynamics as an inherent property of any spatially‐structured landscape (Zeller et al., 2020). And critically, we highlight the fundamental, but often overlooked, role of demography as a major contributor to connectivity dynamics (Drake et al., 2021).

Our four competing parameterizations of a SPOM represents statical analogies of commonly made assumptions in connectivity models. These included (a) assumptions about spatial structure of the system, specifically the inclusion or not of a demographic weighting scheme that explicitly conditions connectivity on the underlying patch occupancy states, and (b) temporal variation in contributions to connectivity, that is, in effective dispersal rates. We note also that the use of a Bayesian hierarchical model allows latent occupancy states to be estimated and thus included in the weighting while still accounting for imperfect detection (Royle & Kery, 2007). These amount to a test of two important components of the quantification of connectivity: refined representations of where dispersers are, and of the dispersal process itself, both of which are inherently dynamic in space and time. We present these results in the context of a classic metapopulation, that is, a highly structured patch network with high rates of dispersal‐driven turnover, which is ideally suited to exploring the consequences of connectivity assumptions. As such, our conclusions, which are likely to hold to various degrees depending on where the system lies on the discrete‐continuous continuum, offer generalities that contribute to a better understanding of the causes and consequences of dynamic connectivity.

An iterative relaxation of assumptions represents a transition of increasing biological realism; here we specifically focus on how demographic contributions to connectivity are introduced. The degree of support for competing formulations of connectivity followed this realism gradient: the UI model (unweighted with time‐invariant dispersal) receiving least support, and the DV model (demographically weighted with time‐varying dispersal) overwhelmingly supported, with models that included a relaxation of either the structural (Dx vs. Ux) or functional (xV vs. xI) falling in between. In our case, relative support for the relaxation of specific assumptions suggests that demographic weighting was more important than allowing for temporally varying dispersal (Table 1). This outcome is notable as the assumptions being relaxed in this study represent those often violated, out of necessity or convenience, in many studies (Drake et al., 2021). In particular, and for example, water voles experience frequent turnover events, limiting the pool of dispersers and introducing false positives in structural measures. The relative importance of specific contributions to connectivity may be inconsistent across systems, wherein depending on landscape heterogeneity and dispersal behaviour, the relative importance of demographics may shift. We emphasize that this does not limit the generality of our approach: the framework we have presented is able to quantify the relative contributions of these two demographic components to connectivity dynamics.

Implicitly assuming homogeneous contributions to connectivity across the landscape does not consider the inherent spatial variation in the distribution of dispersing individuals. In fact, this is akin to a false‐positive observation process in occupancy models, the consequences of which have been described in detail recently (Miller et al., 2011; Royle & Link, 2006). False positives could lead to mis‐estimation of dispersal or colonization ability, extinction rates and a reduction in patch turnover rates (Moilanen, 2002; Sutherland, 2013). For example, even relatively small rates of false positives, that is, designating empty sites as occupied, result in biased inferences about occupancy estimates (Royle & Link, 2006) and occupancy dynamics (Sutherland et al., 2013). Similarly, we find support for our demographically weighted connectivity models that account for such assumptions that create false positives (Table 1; Figure 2). The degree to which this assumption will affect inference is linked to the dependency of spatial dynamics (e.g. occupancy) on dispersal, although we argue that such weighting is necessary in any dispersal dependent systems (Drake et al., 2021).

Connectivity metrics rarely consider temporal dynamics (but see Bishop‐Taylor et al., 2018; Martensen et al., 2017; Ruiz et al., 2014; Hodgson et al., 2009). Our fully spatiotemporally dynamic formulation of a connectivity model allowed for multiple sources of temporal variability, both in the underlying occupancy states and the effective dispersal parameters. What results is substantial variation in effective dispersal (Figure 1), which, in turn, results in variation in estimates of both colonization potential (Figure 2a) and MC (Figure 2b). While individual variability can account for some variation in effective dispersal (Baguette et al., 2013), the spatiotemporal distribution of the disperser pool among habitat patches will likely contribute greatly to the observed variation in effective dispersal. Inclusion of such information into connectivity metrics will better describe observed colonization and occupancy, especially if those population dynamics are thought to be influenced through demographic processes such as the rescue effect (Brown & Kodric‐Brown, 1977), Allee effects (Amarasekare, 1998) or conspecific attraction (Morgan et al., 2019).

As well, environmental shifts (such as climate change) may induce changes in dispersal probability or distances, but these shifts can play out at much different scales (often larger and longer) than demographic processes. Such long‐term processes can influence the structural and functional connectivity between patch networks (Bishop‐Taylor et al., 2018; Drake et al., 2017). In Assynt, there was very little (if any) spatiotemporal variation in the distribution of habitat or the interpatch matrix over the course of the study (Appendix S5). This apparent ‘controlling for’ the potentially confounding influence of spatiotemporal changes in the habitat quality allowed us to isolate the demographic contribution to connectivity. Effective connectivity, connectivity weighted by the effective disperser pool, will likely be driven at shorter scales through populations and by shifts in their dispersal. Local contributions to connectivity, and local connectivity measures, are thus dependent on the location in time and space of conspecifics, as well as the patches they reside in. Furthermore, such explorations of demographically driven dynamic connectivity may require conclusions be made in context of defined temporal windows to account for non‐equilibrium dynamics if equilibrium assumptions may not be made, particularly if spatiotemporal landscape heterogeneity impacts effective dispersal.

Measuring connectivity is difficult, yet the metapopulation paradigm continues to show utility to progress understanding in this regard. Recent attempts to extend the metapopulation paradigm have integrated spatiotemporally variable patch suitability and among‐patch distances, which performed better than static parallels (Bertassello et al., 2021). However, such landscape‐oriented approaches can overlook the demographic or behavioural contributions to connectivity. Indeed, connectivity is an emergent property of demographic processes, for example, dispersal and the spatial distribution of dispersers, which are both spatially and temporally dynamic (Sutherland et al., 2012, 2014). Our model explicitly relaxed such assumptions to allow dispersal to be inferred from population dynamic processes, instead of predefining dispersal with discrete cut‐off values. Such a priori definitions of connections among habitat are often speculative and may misrepresent effective dispersal in the system leading to problematic interpretations of landscape connectivity (Prugh, 2009).

Intraspecific variation in dispersal proclivity and response to external cues also may alter predictions of landscape connectivity and thus be a driver of metapopulation dynamics (Jacob et al., 2019). Dispersal can respond to both external cues and internal phenotype‐dependent factors (Le Galliard et al., 2012) and can vary across the range of a species (Alex Perkins et al., 2013) and over time (Andrew & Ustin, 2010). Such variation may occur through density‐dependent dispersal, phenotypically plastic responses to shifts in individuals environment (Bowler & Benton, 2005), but may also emerge in phenotypically dependent dispersal syndromes which can shift spatiotemporally throughout a population (Clobert et al., 2009; Cote et al., 2017; Fobert et al., 2019). These data are hard to come by, but using the random‐effects structure we adopted for the dispersal model, variability can be captured which can, even in the absence of knowing the mechanism, provide insight into the extent to which connectivity varies, and as such provide important measures of uncertainty that can inform landscape planning, conservation and management, and connectivity science.

MC, although a relative metric, can be sensitive to the scale of dispersal (Blazquez‐Cabrera et al., 2014; Strimas‐Mackey & Brodie, 2018b). Sensitivity analyses are important but uncommon when reporting the MC metric to understand a network's ability to support the metapopulation relative to dispersal capability. Also important, but rarely calculated, is the uncertainty around MC as a point estimate. Uncertainty around key parameters for MC, such as the dispersal rate and scale, propagate and therefore contribute to uncertainty in any derived metric, and MC is no exception. Using the full joint posterior distribution of model parameters, we compared metapopulation capacities of the same network under different assumptions. This showed remarkable variation both in terms of point estimates and associated uncertainty, but demographic weighting resulted in smaller CI than unweighted counterparts for MC estimates. While models incorporating heterogeneity through either demography or dynamism allow for temporal variation to emerge in MC metrics, the static‐unweighted model predicted higher MC with extreme uncertainty (Figure 2b). This lack of variation should not be interpreted as an ‘averaged’ MC; our results suggest that such structurally derived models may consistently misgauge MC alongside the high uncertainty: MC more than halved in some years when considering fully dynamic connectivity relative to static metrics. We stress the need to account for uncertainty in MC; even when accounting for sources of dynamism, there is the potential for erroneous assessment of population persistence and network resilience. Temporal heterogeneity in dispersal matters (Matter et al., 2020) and may be masked by model assumptions or the time series analysed (Ovaskainen & Hanski, 2001; Schnell et al., 2013).

SPOMs, a tool developed to address questions of dynamics in systems assumed to be in long‐term equilibrium or quasi‐equilibrium (Hanski, 2004), have been criticized for their reliance on snap shot data that are either short term or small scale or both (Baguette, 2004). Dispersal estimates may not be accurate if estimated from short time series or small spatial scale (Nathan et al., 2012), potentially over‐ or under‐estimating dispersal rates depending on stochastic variations. Assuming connectivity is static also amounts to estimating long‐term average effective dispersal rates (with the potential that it may not capture a realistic *average* dispersal), overlooking potentially important temporal heterogeneity that can be informative of both demographic and landscape processes impacting population dynamics. This important year‐to‐year variation not only emerges in model parameters, but also in related system‐wide properties (Figure 2); although such variation may not pose a problem to some conservation goals when connectivity or dispersal is not average in one direction, it may be devastating in the other. Thus, focusing on an assumed dispersal capability of a species or population, either via an assumed discrete cut‐off distance, from a snapshot of data, or assumed invariant processes, may not be adequate. Each and all of these may mask short‐term events that can significantly influence long‐term connectivity trends.

Existing approaches to connectivity modelling have been described as often being too naïve or conservative for management reality (Diniz et al., 2020; Nathan et al., 2012). One example of this is the fact that spatiotemporal variation in spatially structured populations can be masked by restrictive model assumptions, precluding the discovery of important underlying variation driving population processes. However, we also recognize that modelling is not the only constraint when approaching connectivity or metapopulation analyses. This specific analysis leveraged a long‐term dataset of generally comprehensive monitoring of the metapopulation. These data allowed us to fit increasing realistic and complex models. Resources to acquire or maintain such datasets are not always available, nor necessary to achieve a specific conservation goal. However, we agree with the general consensus that to solve increasing complex ecological and conservation questions, long‐term monitoring is an essential component (Drake et al., 2021; Lindenmayer & Likens, 2010; Stein et al., 2013).

Our aim here has been to increase awareness about the implications of commonly used modelling decisions on conclusions drawn about a wide range of processes of interest in (meta)population and landscape ecology (e.g. population synchrony, colonization–extinction dynamics, landscape connectivity). In particular, our results support the importance of considering demographic processes as an accounted component of connectivity dynamics (Drake et al., 2021). Indeed, connectivity is dynamic, and we argue via empirical demonstration, that appropriate modelling decisions that link the dynamic process of animal behaviour to the underlying spatial structure of the landscape and the in‐situ populations are essential for accurate characterization and management of connectivity.

## AUTHOR CONTRIBUTIONS

Joseph Drake (JD), Xavier Lambin (XL), and Chris Sutherland (CS) conceived the ideas and designed methodology; J.D., X.L., and C.S. collected the data; J.D. and C.S. analysed the data; J.D. led the writing of the manuscript and all authors contributed critically to the drafts and gave final approval for publication.

## CONFLICT OF INTEREST

There are no conflicts to declare.

## Supporting information


Appendix S1

Appendix S2

Appendix S3

Appendix S4

Appendix S5

## Data Availability

Model output and spatial data are available via the Dryad Digital Repository https://doi.org/10.5061/dryad.sn02v6x70 (Drake et al., 2022). Sensitive location data have been augmented to obscure sensitive species exact locations while retaining relative network structure to allow replicability.
