# Supplementary material for: Spatiotemporal connectivity dynamics in spatially structured populations
Source: J Anim Ecol. 2022 Jul 30;91(10):2050–60. doi: 10.1111/1365-2656.13783 (PMC9796704; doi:10.1111/1365-2656.13783)
Supplement: Supplementary file 1 — Appendix S1 [file JANE-91-2050-s004.pdf]

Appendix S1

Joseph Drake

20220720

Overview

Drake, J. C., Lambin, X., and Sutherland, C. 2022. Spatiotemporal connectivity dynamics in spatially structured populations. Journal of Animal Ecology. DOI: 10.1111/1365-2656.13783

Supplemental information referred to as Appendix S1 in text, including 1) NIMBLE code for dynamic metapopulation code with prior distribution details 2) R code for execution of model, and 3) GVS model selection script

Nimble Dynamic Metapopulation Model

Save this as a separate R script named "nimblecode.R" so that it can be sourced by execution script.

```
###################################################################
#  A Dynamic Col-Ext metapopulation model SPOM
#  Data:
#    Area: a vector of patch sizes
#    dmat: npatch x npatch distance matrix
#    Y: npatch x nyears matrix of detection FREQUENCIES
#    K: npatch x t matrix of number of VISITS
#    z: npatch X nyear matrix of occupancy (1 or NA)
#    nsite: numnber of patches
#    nyear: numnber of years

flexispom <- nimbleCode({

  #~~~~~~~PRIORS~~~~~~~~~~~~~~~~~~~~~~~~~~~~~~~~~~~~~~~~~~~~~~~~~~~~~#

  #PSI1 prior
  psi1 ~ dunif(0,1)

  # Detection prior

  p_mu ~ dnorm(0,0.001)
  p_sd ~ dunif(0,10)
  p_tau <- pow(p_sd, -2)
  for(t in 1:(nyear.obs)){
    P_t[t] ~ dnorm(p_mu, p_tau)
    logit(p_t[t]) <- P_t[t]
```

```
}

# Connectivity model priors

b1_mu ~ dnorm(0, 0.01)
b1_sd ~ dunif(0,10)
b1_tau <- pow(b1_sd, -2)

alpha_mu ~ dnorm(0, 0.01)
alpha_sd ~ dunif(0, 10)
alpha_tau <- pow(alpha_sd, -2)


for(t in 1:(nyear.sim-1)){

  Alpha[t] ~ dnorm(0, alpha_tau)
  alpha[t] <- alpha_mu + c.dyn*Alpha[t]
  sigterm[t] <- 1/(exp(alpha[t]))  # sigterm is mean dispersal distance

  B1_t[t] ~ dnorm(0, b1_tau)
  b1_t[t] <- exp(b1_mu + c.dyn*B1_t[t])

}

# Extinction model priors
# logit(ext) = g0 + g1 * Area
g0_mu ~ dnorm(0, 0.01)
g0_sd ~ dunif(0,10)
g0_tau <- pow(g0_sd, -2)
g1_mu ~ dnorm(0, 0.01)
g1_sd ~ dunif(0,10)
g1_tau <- pow(g0_sd, -2)

#time specific random transition parameters
for(t in 1:(nyear.sim-1)){
  G0_t[t] ~ dnorm(0, g0_tau)
  G1_t[t] ~ dnorm(0, g1_tau)
  g0_t[t] <- g0_mu + e.dyn*G0_t[t]
  g1_t[t] <- g1_mu + e.dyn*G1_t[t]
}


#~~~~~~~Likelihood~~~~~~~~~~~~~~~~~~~~~~~~~~~~~~~~~~~~~~~~~~~~~~~~~~~~#

for(i in 1:nsite){           #initial occupancy t0
  z[i,1] ~ dbern(psi1)
}

for(k in 2:nyear.sim){       #for occupancy t1 and after
```

```
    for(i in 1:nsite){
      for(j in 1:nsite){
        con[i,j,k-1] <- exp(-sigterm[k-1] * dmat[i,j]) * #kernel
                        (1 - equals(i,j)) *             #self
                        max(z[j,k-1], struct) *         #functional weight
                        Area[j]                         #area weight contrib
      }

      #transition probs
      conx[i,k-1] <- sum(con[i,1:nsite,k-1])

      col[i,k-1] <- 1-exp(-b1_t[k-1]*conx[i,k-1]) #  akin to Sutherland et al
. 2014 to help with model convergence
      logit(ext[i,k-1]) <- g0_mu + g1_mu * Area[i]

      #occupancy
      mu.z[i,k-1] <- z[i,k-1] * max(0.001, min((1-ext[i,k-1]), 0.999)) +
                     (1 - z[i,k-1]) * max(0.001, min(col[i,k-1],  0.999)) # m
in-max trick to prevent calculation issues
      z[i,k] ~ dbern(mu.z[i,k-1])
    }
  }
  #### observation model
  for(i in 1:nsite){
    for (t in 1:nyear.obs){
      mu.p[i, t] <- z[i,t] * p_t[t]
      Y[i, t] ~ dbin(mu.p[i, t], K[i,t])
    }
  }
  #### Derived parameters
  for(t in 1:nyear.sim){
    m.occ[t] <- sum(z[1:nsite,t])
    }

})
```

R Script for Model Execution

```
library(nimble)

load("Data.RData") # this is a place holder for data described below


#  Data:
#    Area: a vector of patch sizes
#    dmat: npatch x npatch distance matrix
#    Y: npatch x nyears matrix of detection FREQUENCIES
#    K: npatch x t matrix of number of VISITS
#    z: npatch X nyear matrix of occupancy (1 or NA)
#    nsite: numnber of patches
```

```
#    nyear: numnber of years




data <- list(Area=Area, #
             Y=Y,       #
             K=K,       #
             dmat=dmat, #
             z=z)       #

#1. struct. connectivity (nwork position only) + static effect (beta_t = beta
)  (model UI)
#2. struct. connectivity (nwork position only) + dynamic effect (beta_t)
(model UV)
#3. funct. connectivity (z-weighted) + static effect (beta_t = beta)
(model DI)
#4. funct. connectivity (z-weighted) + dynamic effect (beta_t)
(model DV)

#1 and 2 are *non*-demographic or demographically naive
#3 and 4 are demographic connectivity

#model 1
sta.consts.struct <- list(nyear.obs=nyear.obs,
                   nyear.sim=nyear.sim,
                   nsite=nsite,
                   c.dyn=0,  #0=invariant, 1=time-varying
                   e.dyn=0,  #0=invariant, 1=time-varying
                   struct=1) #1=structural, 0=functional

#model 2
dyn.consts.struct <- list(nyear.obs=nyear.obs,
                   nyear.sim=nyear.sim,
                   nsite=nsite,
                   c.dyn=1,
                   e.dyn=0,
                   struct=1) #1=structural, 0=functional

#model 3
sta.consts <- list(nyear.obs=nyear.obs,
                   nyear.sim=nyear.sim,
                   nsite=nsite,
                   c.dyn=0,
                   e.dyn=0,
                   struct=0) #1=structural, 0=functional

#model 4
dyn.consts <- list(nyear.obs=nyear.obs,
```

```
                   nyear.sim=nyear.sim,
                   nsite=nsite,
                   c.dyn=1,
                   e.dyn=0,
                   struct=0) #1=structural, 0=functional


# Parameters to track
params <- c("alpha","b1_t","m.occ","sigterm", "Alpha", "alpha_mu", "b1_mu", "
B1_t")


inits <- function(){
    list( psi1=runif(1,0.1,0.9),
          p_mu=rnorm(1,0,0.1),
          p_sd=runif(1,0.1,1),
          P_t=rnorm(nyear.obs,0,0.1),

          alpha_mu=rnorm(1,0,0.1),
          alpha_sd=runif(1,0.1,1),
          b1_mu=rnorm(1,0,0.1),
          b1_sd=runif(1,0.1,1),
          B1_t=rnorm(nyear.sim-1,0,0.1),

          g0_mu=runif(1,-1,1),
          g1_mu=rnorm(1,-1,0.1),
          G0_t=rnorm(nyear.sim-1,0,0.1),
          G1_t=rnorm(nyear.sim-1,0,0.1))
}

source("nimblecode.R")


mp_DV <- nimbleMCMC(code=flexispom,
                         constants=dyn.consts,
                         data=data, inits=inits, monitors = params,
                         nchains=3, niter = 80000, nburnin = 30000,    # 80k r
un 30k burnin
                         thin = 1, summary = TRUE, WAIC =FALSE,
                         check= TRUE, samples = TRUE, samplesAsCodaMCMC=TRUE)
save(mp_DV, file=paste("mp_DV",format(Sys.time(), "%Y%m%d"), ".RData", sep=""
))


mp_UV <- nimbleMCMC(code=flexispomv,
                         constants=dyn.consts.struct,
                         data=data, inits=inits, monitors = params,
                         nchains=3, niter = 80000, nburnin = 30000,
                         thin = 1, summary = TRUE, WAIC = FALSE,
```

```
                         check= TRUE, samples = TRUE, samplesAsCodaMCMC=TRUE)
save(mp_UV, file=paste("mp_UV",format(Sys.time(), "%Y%m%d"), ".RData", sep=""
))


mp_DI  <- nimbleMCMC(code=flexispom,
                         constants=sta.consts,
                         data=data, inits=inits, monitors = params,
                         nchains=3, niter = 80000, nburnin = 30000,
                         thin = 1, summary = TRUE, WAIC = FALSE,
                         check= TRUE, samples = TRUE, samplesAsCodaMCMC=TRUE)
save(mp_DI, file=paste("mp_DI",format(Sys.time(), "%Y%m%d"), ".RData", sep=""
))


mp_UI  <- nimbleMCMC(code=flexispom,
                         constants=sta.consts.struct,
                         data=data, inits=inits, monitors = params,
                         nchains=3, niter = 80000, nburnin = 30000,
                         thin = 1, summary = TRUE, WAIC = FALSE,        # Use
params2 for WAIC=TRUE
                         check= TRUE, samples = TRUE, samplesAsCodaMCMC=TRUE)
save(mp_UI, file=paste("mp_UI",format(Sys.time(), "%Y%m%d"), ".RData", sep=""
))
```

Nimble GVS model selection Script

Save this as a separate R script named “gvscode.R” so that it can be sourced by execution script.

```
modelselection <- nimbleCode({

  #~~~~~~~PRIORS~~~~~~~~~~~~~~~~~~~~~~~~~~~~~~~~~~~~~~~~~~~~~~~~~~~~~~~~~~#

  #PSI1 prior
  psi1 ~ dunif(0,1)

  pick ~ dcat(probs[1:4])

  mod <- equals(pick,1) * 1  +
         equals(pick,2) * 2  +
         equals(pick,3) * 3  +
         equals(pick,4) * 4


  structural <- mod.binary[mod,1] #
  c.dyn  <- mod.binary[mod,2]

  #detection prior
```

```
  p_mu ~ dnorm(0,0.001)
  p_sd ~ dunif(0,10)
  p_tau <- pow(p_sd, -2)
  for(t in 1:(nyear.obs)){
    P_t[t] ~ dnorm(p_mu, p_tau)
    logit(p_t[t]) <- P_t[t]
  }

  #####connectivity model priors
  b1_mu ~ dnorm(0, 0.01)
  b1_sd ~ dunif(0,10)
  b1_tau <- pow(b1_sd, -2)

  alpha_mu ~ dnorm(0, 0.01)
  alpha_sd ~ dunif(0, 10)
  alpha_tau <- pow(alpha_sd, -2)

 for(t in 1:(nyear.sim-1)){

    Alpha[t] ~ dnorm(0, alpha_tau)
    alpha[t] <- alpha_mu + c.dyn*Alpha[t]
    sigterm[t] <- 1/(exp(alpha[t]))

    B1_t[t] ~ dnorm(0, b1_tau)
    b1_t[t] <- exp(b1_mu + c.dyn*B1_t[t])

  }

  # Extinction model priors
  g0_mu ~ dnorm(0, 0.01)
  g0_sd ~ dunif(0,10)
  g0_tau <- pow(g0_sd, -2)
  g1_mu ~ dnorm(0, 0.01)
  g1_sd ~ dunif(0,10)
  g1_tau <- pow(g0_sd, -2)

  #time specific random transition parameters
  for(t in 1:(nyear.sim-1)){
    G0_t[t] ~ dnorm(0, g0_tau)
    G1_t[t] ~ dnorm(0, g1_tau)
    g0_t[t] <- g0_mu + e.dyn*G0_t[t]
    g1_t[t] <- g1_mu + e.dyn*G1_t[t]
  }


  #~~~~~~~Likelihood~~~~~~~~~~~~~~~~~~~~~~~~~~~~~~~~~~~~~~~~~~~~~~~~~#

  for(i in 1:nsite){           #initial occupancy t0
    z[i,1] ~ dbern(psi1)
```

```
  }

  for(k in 2:nyear.sim){      #for occupancy t1 and after
    for(i in 1:nsite){
      for(j in 1:nsite){
        con[i,j,k-1] <- exp(-sigterm[k-1] * dmat[i,j]) * #kernel
          (1 - equals(i,j)) *                           #self
          max(z[j,k-1], structural) *                   #functional weight
          Area[j]                                       #area weight contrib
      }

      #transition probs
      conx[i,k-1] <- sum(con[i,1:nsite,k-1])
      col[i,k-1] <- 1-exp(-b1_t[k-1]*conx[i,k-1]) #  akin to Sutherland et al
. 2014 to help with model convergence
      logit(ext[i,k-1]) <- g0_mu + g1_mu * Area[i]

      #occupancy
      mu.z[i,k-1] <- z[i,k-1] * max(0.001, min((1-ext[i,k-1]), 0.999)) +
        (1 - z[i,k-1]) * max(0.001, min(col[i,k-1],  0.999))
      z[i,k] ~ dbern(mu.z[i,k-1])
    }
  }
  #### observation model
  for(i in 1:nsite){
    for (t in 1:nyear.obs){
      mu.p[i, t] <- z[i,t] * p_t[t]
      Y[i, t] ~ dbin(mu.p[i, t], K[i,t])
    }
  }
  #### Derived parameters
  for(t in 1:nyear.sim){
    m.occ[t] <- sum(z[1:nsite,t])
  }
})
```

GVS model selection R script

```
library(nimble)


#  Data:
#    Area: a vector of patch sizes
#    dmat: npatch x npatch distance matrix
#    Y: npatch x nyears matrix of detection FREQUENCIES
#    K: npatch x t matrix of number of VISITS
#    z: npatch X nyear matrix of occupancy (1 or NA)
#    nsite: numnber of patches
#    nyear: numnber of years
```

```
## Model selection matrix

# column 1 = func (0) vs. struc (1)
# column 2 = dynamic = 1, static =0
mod.binary <- matrix(c(1,0,               # struc static  (model UI)
                       1,1,               # struc dynamic (model UV)
                       0,0,               # func  static  (model DI)
                       0,1), 4,2, byrow=TRUE)  # func dynamic (model DV)


#1. struct. connectivity (nwork position only) + static effect (beta_t = beta
)  (model UI)
#2. struct. connectivity (nwork position only) + dynamic effect (beta_t)
(model UV)
#3. funct. connectivity (z-weighted) + static effect (beta_t = beta)
(model DI)
#4. funct. connectivity (z-weighted) + dynamic effect (beta_t)
(model DV)

#1 and 2 are *non*-demographic or demographically naive
#3 and 4 are demographic connectivity

data <- list(mod.binary=mod.binary,
             probs=c(0.25,0.25,0.25,0.25),
             Area=Area,
             Y=Y,
             K=K,
             dmat=dmat,
             z=z)



#constants

consts <- list(    mods=as.numeric(c(1,2,3,4)), # the list of models referenc
ed above
                   nyear.obs=nyear.obs, # number of years in data
                   nyear.sim=nyear.sim,
                   nsite=nsite,         # number of sites in data
                   e.dyn=0
                   )


params <- c("pick") # the parameter to track, which shows which model is sele
cted by the GVS process



inits <- function(){
```

```
  list( psi1=runif(1,0.1,0.9),
        pick=1,
        p_mu=rnorm(1,0,0.1),
        p_sd=runif(1,0.1,1),
        P_t=rnorm(nyear.obs,0,0.1),

        alpha_mu=rnorm(1,0,0.1),
        alpha_sd=runif(1,0.1,1),
        b1_mu=rnorm(1,0,0.1),
        b1_sd=runif(1,0.1,1),
        B1_t=rnorm(nyear.sim-1,0,0.1),

        g0_mu=runif(1,-1,1),
        g1_mu=rnorm(1,-1,0.1),
        G0_t=rnorm(nyear.sim-1,0,0.1),
        G1_t=rnorm(nyear.sim-1,0,0.1))
}

source("gvs.R")


mp_modelselect <- nimbleMCMC(code=modelselection,
                         constants=consts,
                         data=data, inits=inits2, monitors = params,
                         nchains=3, niter = 110000, nburnin = 10000,
                         thin = 1, summary = TRUE, WAIC = FALSE,
                         check= TRUE, samples = TRUE, samplesAsCodaMCMC=TRUE)
save(mp_modelselect, file=paste("mp_modselect",format(Sys.time(), "%Y%m%d"),
".RData", sep=""))
```
