# Supplementary material for: Spatiotemporal connectivity dynamics in spatially structured populations
Source: J Anim Ecol. 2022 Jul 30;91(10):2050–60. doi: 10.1111/1365-2656.13783 (PMC9796704; doi:10.1111/1365-2656.13783)
Supplement: Supplementary file 2 — Appendix S2 [file JANE-91-2050-s001.docx]

Drake, J. C., Lambin, X., and Sutherland, C. 2022. Spatiotemporal connectivity dynamics in spatially structured populations. Journal of Animal Ecology. DOI: 10.1111/1365-2656.13783

Appendix S2: This appendix contains supplemental information in support of results from Drake et al. (2022). These results are parameter and occupancy estimates for 4 different models assuming various degrees of realism for the connectivity process.

Table S2A. Parameter estimates for four dynamic and spatially-explicit stochastic patch occupancy models. Each model represents various amounts of abstraction for connectivity in the landscape: demographically-weighted and time-varying (DV), unweighted and time-varying (UV), demographically-weighted and time-invariant (DI), & unweighted and time-invariant (UI). Here α - µ represents the raw parameter estimate of the random effects mean of the dispersal scaling factor, while α – 1999 to α – 2015 represents year specific parameter estimate. Notice that time-invariant models do not change. The link function to bring this to kilometers would be 1/e^α^ to represent mean dispersal distance. βt - µ represents the random effects mean of the connectivity parameter representing the per capita effective rate of dispersal. βt – 1999 to βt – 2015 represents the year specific value of the per capita rate of dispersal. Also note that time-invariant models do not vary across time. LCI and UCI stand for the lower confidence interval and upper confidence interval respectively.

| Parameter | DV Posterior mean | (95% LCI) | (95% UCI) | UV Posterior mean | (95% LCI) | (95% UCI) | DI Posterior mean | (95% LCI) | (95% UCI) | UI Posterior mean | (95% LCI) | (95% UCI) |
| --- | --- | --- | --- | --- | --- | --- | --- | --- | --- | --- | --- | --- |
| α - µ | 0.531 | -0.080 | 1.153 | 0.657 | -0.042 | 1.559 | 0.846 | 0.304 | 1.444 | 0.863 | 0.127 | 1.764 |
|  |  |  |  |  |  |  |  |  |  |  |  |  |
| α - 1999 | 0.522 | -0.374 | 1.316 | 0.635 | -0.348 | 1.673 | - | - | - | - | - | - |
| α - 2000 | 0.447 | -0.558 | 1.284 | 0.596 | -0.467 | 1.706 | - | - | - | - | - | - |
| α - 2001 | 0.619 | -0.207 | 1.573 | 0.768 | -0.204 | 2.032 | - | - | - | - | - | - |
| α - 2002 | 0.390 | -0.783 | 1.242 | 0.483 | -0.705 | 1.553 | - | - | - | - | - | - |
| α - 2003 | 0.484 | -0.344 | 1.274 | 0.604 | -0.340 | 1.674 | - | - | - | - | - | - |
| α - 2004 | 0.445 | -0.378 | 1.197 | 0.525 | -0.460 | 1.527 | - | - | - | - | - | - |
| α - 2005 | 0.616 | 0.026 | 1.265 | 0.691 | -0.040 | 1.589 | - | - | - | - | - | - |
| α - 2006 | 0.736 | 0.061 | 1.587 | 0.886 | 0.055 | 2.179 | - | - | - | - | - | - |
| α - 2007 | 0.485 | -0.384 | 1.278 | 0.594 | -0.412 | 1.634 | - | - | - | - | - | - |
| α - 2008 | 0.460 | -0.546 | 1.296 | 0.513 | -0.582 | 1.538 | - | - | - | - | - | - |
| α - 2009 | 0.596 | -0.056 | 1.320 | 0.690 | -0.043 | 1.637 | - | - | - | - | - | - |
| α - 2010 | 0.599 | -0.148 | 1.412 | 0.757 | -0.098 | 1.915 | - | - | - | - | - | - |
| α - 2011 | 0.455 | -0.465 | 1.258 | 0.582 | -0.392 | 1.599 | - | - | - | - | - | - |
| α – 2012 | 0.564 | -0.280 | 1.444 | 0.721 | -0.159 | 1.873 | - | - | - | - | - | - |
| α – 2013 | 0.643 | -0.190 | 1.555 | 0.891 | 0.013 | 2.273 | - | - | - | - | - | - |
| α – 2014 | 0.471 | -0.433 | 1.287 | 0.598 | -0.406 | 1.686 | - | - | - | - | - | - |
| α – 2015 | 0.505 | -0.304 | 1.273 | 0.671 | -0.198 | 1.706 | - | - | - | - | - | - |
|  |  |  |  |  |  |  |  |  |  |  |  |  |
| βt - µ | -1.938 | -2.922 | -0.947 | -2.441 | -3.612 | -1.418 | -2.135 | -2.883 | -1.351 | -2.473 | -3.511 | -1.471 |
|  |  |  |  |  |  |  |  |  |  |  |  |  |
| βt - 1999 | 0.134 | 0.016 | 0.416 | 0.090 | 0.012 | 0.277 | - | - | - | - | - | - |
| βt - 2000 | 0.134 | 0.012 | 0.494 | 0.091 | 0.009 | 0.324 | - | - | - | - | - | - |
| βt - 2001 | 0.299 | 0.049 | 1.040 | 0.166 | 0.024 | 0.562 | - | - | - | - | - | - |
| βt - 2002 | 0.099 | 0.010 | 0.347 | 0.074 | 0.008 | 0.260 | - | - | - | - | - | - |
| βt - 2003 | 0.161 | 0.027 | 0.514 | 0.082 | 0.013 | 0.248 | - | - | - | - | - | - |
| βt - 2004 | 0.125 | 0.022 | 0.392 | 0.066 | 0.010 | 0.208 | - | - | - | - | - | - |
| βt - 2005 | 0.209 | 0.057 | 0.547 | 0.105 | 0.025 | 0.275 | - | - | - | - | - | - |
| βt - 2006 | 0.602 | 0.128 | 1.808 | 0.282 | 0.055 | 0.804 | - | - | - | - | - | - |
| βt - 2007 | 0.137 | 0.016 | 0.454 | 0.091 | 0.010 | 0.300 | - | - | - | - | - | - |
| βt - 2008 | 0.085 | 0.014 | 0.268 | 0.059 | 0.010 | 0.190 | - | - | - | - | - | - |
| βt - 2009 | 0.206 | 0.064 | 0.505 | 0.114 | 0.030 | 0.287 | - | - | - | - | - | - |
| βt - 2010 | 0.461 | 0.118 | 1.359 | 0.273 | 0.055 | 0.801 | - | - | - | - | - | - |
| βt - 2011 | 0.140 | 0.023 | 0.458 | 0.100 | 0.016 | 0.325 | - | - | - | - | - | - |
| βt – 2012 | 0.156 | 0.036 | 0.457 | 0.103 | 0.020 | 0.303 | - | - | - | - | - | - |
| βt – 2013 | 0.588 | 0.132 | 1.915 | 0.313 | 0.059 | 0.924 | - | - | - | - | - | - |
| βt – 2014 | 0.118 | 0.016 | 0.391 | 0.087 | 0.012 | 0.283 | - | - | - | - | - | - |
| βt - 2015 | 0.214 | 0.047 | 0.658 | 0.131 | 0.026 | 0.395 | - | - | - | - | - | - |

Table S2B. Mean posterior estimates for occupancy as fraction of the 98 patches in the Assynt metapopulation system for four dynamic and spatially-explicit stochastic patch occupancy models. Each model represents various amounts of abstraction for connectivity in the landscape: demographically-weighted and time-varying (DV), unweighted and time-varying (UV), demographically-weighted and time-invariant (DI), & unweighted and time-invariant (UI). LCI and UCI stand for the lower confidence interval and upper confidence interval respectively.

| Occupancy | DV Posterior mean | (95% LCI) | (95% UCI) | UV Posterior mean | (95% LCI) | (95% UCI) | DI Posterior mean | (95% LCI) | (95% UCI) | UI Posterior mean | (95% LCI) | (95% UCI) |
| --- | --- | --- | --- | --- | --- | --- | --- | --- | --- | --- | --- | --- |
| 1999 | 0.698 | 0.551 | 0.888 | 0.704 | 0.551 | 0.898 | 0.646 | 0.531 | 0.837 | 0.666 | 0.541 | 0.867 |
| 2000 | 0.650 | 0.551 | 0.765 | 0.658 | 0.561 | 0.776 | 0.651 | 0.561 | 0.755 | 0.667 | 0.571 | 0.765 |
| 2001 | 0.603 | 0.469 | 0.786 | 0.626 | 0.490 | 0.806 | 0.654 | 0.561 | 0.745 | 0.667 | 0.571 | 0.755 |
| 2002 | 0.676 | 0.582 | 0.796 | 0.680 | 0.582 | 0.816 | 0.640 | 0.571 | 0.724 | 0.659 | 0.582 | 0.745 |
| 2003 | 0.576 | 0.480 | 0.684 | 0.594 | 0.500 | 0.704 | 0.597 | 0.510 | 0.684 | 0.628 | 0.541 | 0.714 |
| 2004 | 0.564 | 0.490 | 0.653 | 0.575 | 0.500 | 0.663 | 0.578 | 0.500 | 0.663 | 0.611 | 0.531 | 0.704 |
| 2005 | 0.548 | 0.490 | 0.622 | 0.552 | 0.490 | 0.633 | 0.599 | 0.531 | 0.684 | 0.619 | 0.541 | 0.704 |
| 2006 | 0.662 | 0.602 | 0.735 | 0.664 | 0.602 | 0.735 | 0.708 | 0.643 | 0.776 | 0.715 | 0.653 | 0.786 |
| 2007 | 0.888 | 0.847 | 0.929 | 0.892 | 0.847 | 0.929 | 0.864 | 0.827 | 0.898 | 0.867 | 0.837 | 0.898 |
| 2008 | 0.625 | 0.571 | 0.694 | 0.620 | 0.571 | 0.684 | 0.598 | 0.551 | 0.653 | 0.604 | 0.561 | 0.663 |
| 2009 | 0.553 | 0.510 | 0.602 | 0.554 | 0.510 | 0.602 | 0.570 | 0.531 | 0.622 | 0.572 | 0.520 | 0.633 |
| 2010 | 0.633 | 0.612 | 0.663 | 0.633 | 0.612 | 0.663 | 0.638 | 0.612 | 0.673 | 0.639 | 0.612 | 0.673 |
| 2011 | 0.855 | 0.837 | 0.878 | 0.855 | 0.837 | 0.878 | 0.849 | 0.827 | 0.867 | 0.850 | 0.827 | 0.867 |
| 2012 | 0.656 | 0.592 | 0.724 | 0.658 | 0.592 | 0.724 | 0.665 | 0.602 | 0.735 | 0.668 | 0.602 | 0.735 |
| 2013 | 0.668 | 0.612 | 0.735 | 0.672 | 0.612 | 0.745 | 0.725 | 0.663 | 0.786 | 0.722 | 0.653 | 0.786 |
| 2014 | 0.920 | 0.908 | 0.939 | 0.921 | 0.908 | 0.939 | 0.917 | 0.908 | 0.929 | 0.917 | 0.908 | 0.929 |
| 2015 | 0.775 | 0.724 | 0.827 | 0.780 | 0.724 | 0.837 | 0.776 | 0.724 | 0.827 | 0.782 | 0.735 | 0.837 |
| 2016 | 0.731 | 0.694 | 0.776 | 0.732 | 0.694 | 0.776 | 0.733 | 0.694 | 0.776 | 0.734 | 0.694 | 0.776 |
