# Supplementary material for: Spatiotemporal connectivity dynamics in spatially structured populations
Source: J Anim Ecol. 2022 Jul 30;91(10):2050–60. doi: 10.1111/1365-2656.13783 (PMC9796704; doi:10.1111/1365-2656.13783)
Supplement: Supplementary file 3 — Appendix S3 [file JANE-91-2050-s003.pdf]

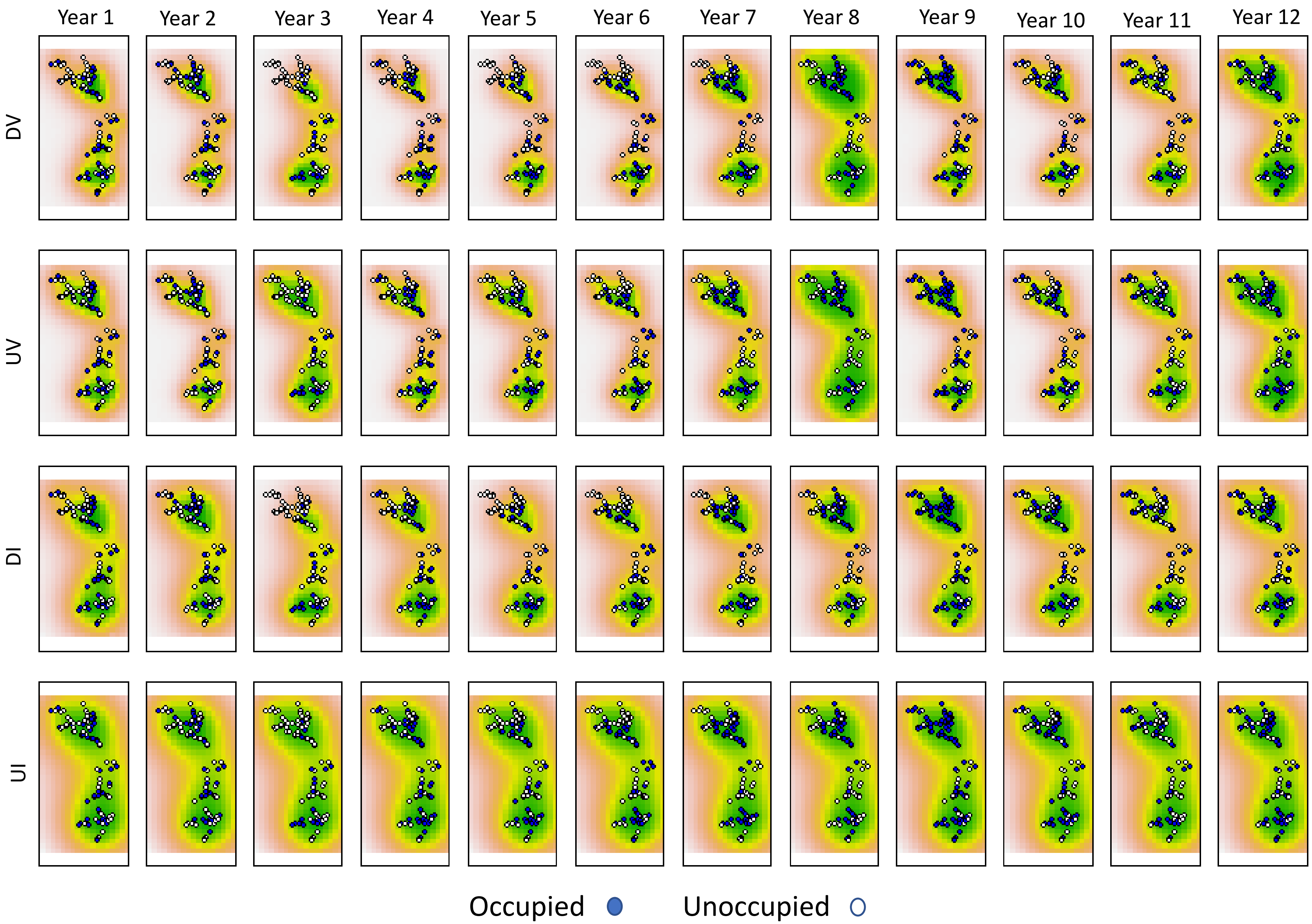

Appendix S3.

Figure S3A. This figure represents the realized connectivity between habitat patches across all 12 scenario years. Demographically-weighted and time-varying connectivity (DV), unweighted and time-varying connectivity (UV), & demographically-weighted and time-invariant connectivity (DI) all contain varying levels of dynamism either accounted through model structure or through the inclusion of demographic data in the form of occupancy state-variables. Unweighted with time-invariant connectivity (UI) shows that no matter the observed occupancy in the landscape, the connectivity fails to shift, representing a structural view of the connectivity in the landscape. DV was the most supported connectivity model based on Bayesian model selection and the model UI was least supported.

Drake, J. C., Lambin, X., and Sutherland, C. 2022. Spatiotemporal connectivity dynamics in spatially structured populations. Journal of Animal Ecology. DOI: 10.1111/1365- 2656.13783

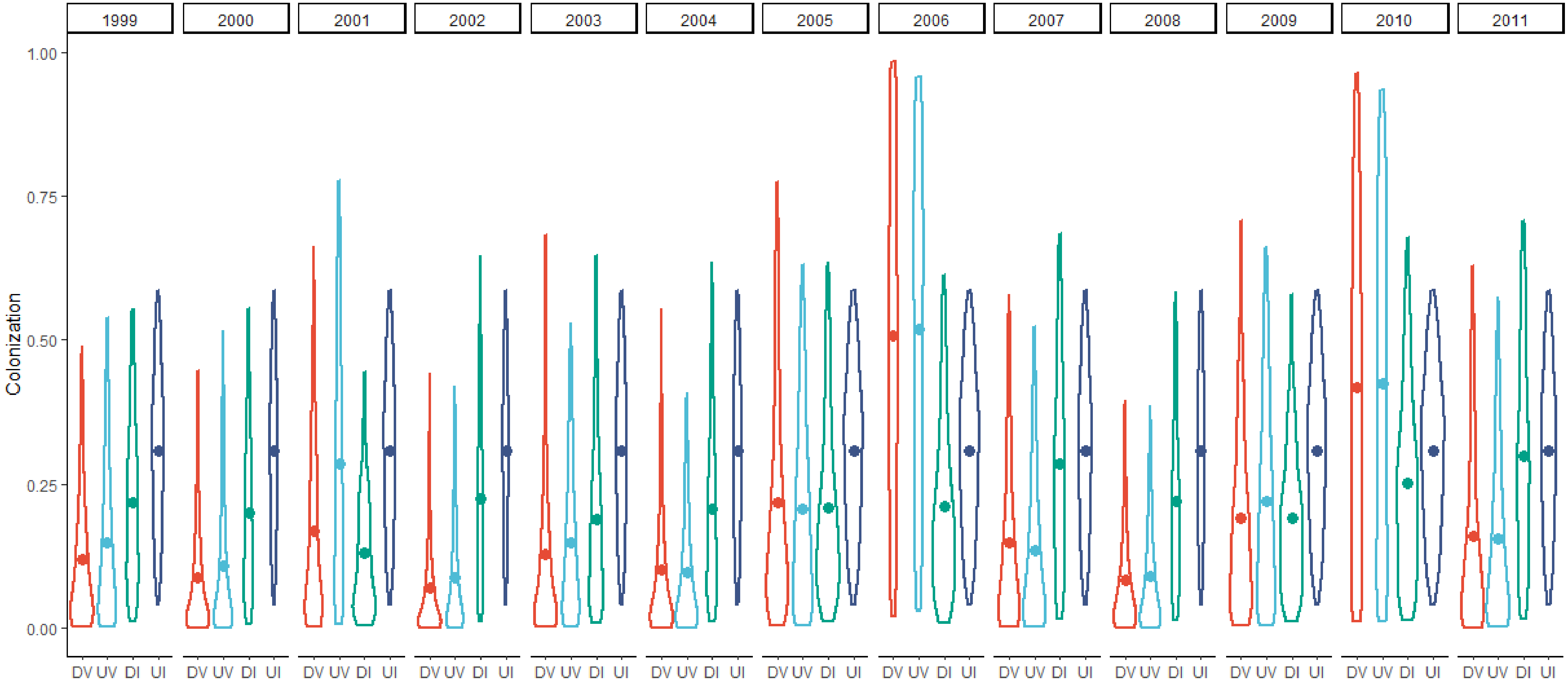

Appendix S3.

Figure S3B. This figure represents the distribution and range of realized landscape pixel values among patches across all years. Dots represent the mean landscape colonization potential for demographically-weighted and time-varying connectivity (DV), unweighted and time-varying connectivity (UV), & demographically-weighted and time-invariant connectivity (DI). This is similar to Figure 2A in text but includes a visualization of the distribution of landscape pixel values to illustrate differences between models.

Drake, J. C., Lambin, X., and Sutherland, C. 2022. Spatiotemporal connectivity dynamics in spatially structured populations. Journal of Animal Ecology. DOI: 10.1111/1365- 2656.13783
