# Supplementary material for: Spatiotemporal connectivity dynamics in spatially structured populations
Source: J Anim Ecol. 2022 Jul 30;91(10):2050–60. doi: 10.1111/1365-2656.13783 (PMC9796704; doi:10.1111/1365-2656.13783)
Supplement: Supplementary file 4 — Appendix S4 [file JANE-91-2050-s002.docx]

Appendix S4

Joseph Drake

06/07/2022

## Overview

Drake, J. C., Lambin, X., and Sutherland, C. 2022. Spatiotemporal connectivity dynamics in spatially structured populations. Journal of Animal Ecology. DOI: 10.1111/1365- 2656.13783

Supplemental information referred to as Appendix S4 in text, including NIMBLE code for dynamic metapopulation code with prior distribution details and GoF test statistic calculation

## Nimble Dynamic Metapopulation Model

Save this as a separate R script named “nimblecode.R” so that it can be sourced by execution script.

###################################################################
# A Col-Ext metapopulation Goodness-of-Fit Freeman Tukey test model
# Data:
# Area: a vector of patch sizes
# dmat: npatch x npatch distance matrix
# Y: npatch x nyears matrix of detection FREQUENCIES
# K: npatch x t matrix of number of VISITS
# nsite: numnber of patches
# nyear: numnber of years

SPOM_GoF <- nimbleCode({

 #~~~~~~~PRIORS~~~~~~~~~~~~~~~~~~~~~~~~~~~~~~~~~~~~~~~~~~~~~~~~~~~~~#

 #PSI1 prior
 psi1 ~ dunif(0,1)

 #detection prior
 p_mu ~ dnorm(0,0.001)
 p_sd ~ dunif(0,10)
 p_tau <- pow(p_sd, -2)
 for(t in 1:(nyear.obs)){
 P_t[t] ~ dnorm(p_mu, p_tau)
 logit(p_t[t]) <- P_t[t]
 }

 #####connectivity model priors

 b1_mu ~ dnorm(0, 0.01)
 b1_sd ~ dunif(0,10)
 b1_tau <- pow(b1_sd, -2)

 alpha_mu ~ dnorm(0, 0.01)
 alpha_sd ~ dunif(0, 10)
 alpha_tau <- pow(alpha_sd, -2)


 for(t in 1:(nyear.sim-1)){

 Alpha[t] ~ dnorm(0, alpha_tau)
 alpha[t] <- alpha_mu + c.dyn*Alpha[t]
 sigterm[t] <- 1/(exp(alpha[t]))

 B1_t[t] ~ dnorm(0, b1_tau)
 b1_t[t] <- exp(b1_mu + c.dyn*B1_t[t])

 }


 #extinction model priors
 # logit(ext) = g0 + g1 * Area
 g0_mu ~ dnorm(0, 0.01)
 g0_sd ~ dunif(0,10)
 g0_tau <- pow(g0_sd, -2)
 g1_mu ~ dnorm(0, 0.01)
 g1_sd ~ dunif(0,10)
 g1_tau <- pow(g0_sd, -2)

 #time specific random transition parameters
 for(t in 1:(nyear.sim-1)){
 G0_t[t] ~ dnorm(0, g0_tau)
 G1_t[t] ~ dnorm(0, g1_tau)
 g0_t[t] <- g0_mu + e.dyn*G0_t[t]
 g1_t[t] <- g1_mu + e.dyn*G1_t[t]
 }


 #~~~~~~~Likelihood~~~~~~~~~~~~~~~~~~~~~~~~~~~~~~~~~~~~~~~~~~~~~~~~~#

 for(i in 1:nsite){ #initial occupancy t0
 z[i,1] ~ dbern(psi1)
 }

 for(k in 2:nyear.sim){ #for occupancy t1 and after
 for(i in 1:nsite){
 for(j in 1:nsite){
 con[i,j,k-1] <- exp(-sigterm[k-1] * dmat[i,j]) * #kernel
 (1 - equals(i,j)) * #self
 max(z[j,k-1], struct) * #functional weight
 Area[j] #area weight contrib
 }

 #transition probs
 conx[i,k-1] <- sum(con[i,1:nsite,k-1])
 #logit(col[i,k-1]) <- b0_t[k-1] + b1_t[k-1] * conx[i,k-1]
 col[i,k-1] <- 1-exp(-b1_t[k-1]*conx[i,k-1]) # akin to Sutherland et al. 2014 to help with model convergence
 logit(ext[i,k-1]) <- g0_mu + g1_mu * Area[i]

 #occupancy
 mu.z[i,k-1] <- z[i,k-1] * max(0.001, min((1-ext[i,k-1]), 0.999)) +
 (1 - z[i,k-1]) * max(0.001, min(col[i,k-1], 0.999))
 z[i,k] ~ dbern(mu.z[i,k-1])
 }
 }
 #### observation model
 for(i in 1:nsite){
 for (t in 1:nyear.obs){
 mu.p[i, t] <- z[i,t] * p_t[t]
 Y[i, t] ~ dbin(mu.p[i, t], K[i,t])

 # ## observation level GOF stuff ###############################################


 # sim & observed data
 yrep[i,t] ~ dbin(mu.p[i,t]*z[i,t], K[i,t]) # or K[i,t]? Also is it p_t[t]?
 yexp[i,t] <- mu.p[i,t]*K[i,t]*z[i,t] + 0.001 # or K[i,t]? Just p_t?

 #Freeman-tukeys
 x2.obs[i,t] <- pow((sqrt(Y[i,t]) - sqrt(yexp[i,t])), 2)
 x2.sim[i,t] <- pow((sqrt(yrep[i,t]) - sqrt(yexp[i,t])), 2)

 }
 }

 ## sum over observation ############################################
 chi2.obs <- sum(x2.obs[1:nsite,1:nyear.obs]) # sum over observations outside the loop
 chi2.rep <- sum(x2.sim[1:nsite,1:nyear.obs]) # sum over replicates outside the loop

 #### Derived parameters
 for(t in 1:nyear.sim){
 m.occ[t] <- sum(z[1:nsite,t])

 }

})
