# Supplementary material for: Spatiotemporal connectivity dynamics in spatially structured populations
Source: J Anim Ecol. 2022 Jul 30;91(10):2050–60. doi: 10.1111/1365-2656.13783 (PMC9796704; doi:10.1111/1365-2656.13783)
Supplement: Supplementary file 5 — Appendix S5 [file JANE-91-2050-s005.docx]

Drake, J. C., Lambin, X., and Sutherland, C. 2022. Spatiotemporal connectivity dynamics in spatially structured populations. Journal of Animal Ecology. DOI: 10.1111/1365- 2656.13783

Appendix S5

Landcover change in Assynt between 2019 and 2020 based off satellite imagery derived landcover classification of Scotland (Space Intelligence 2021).


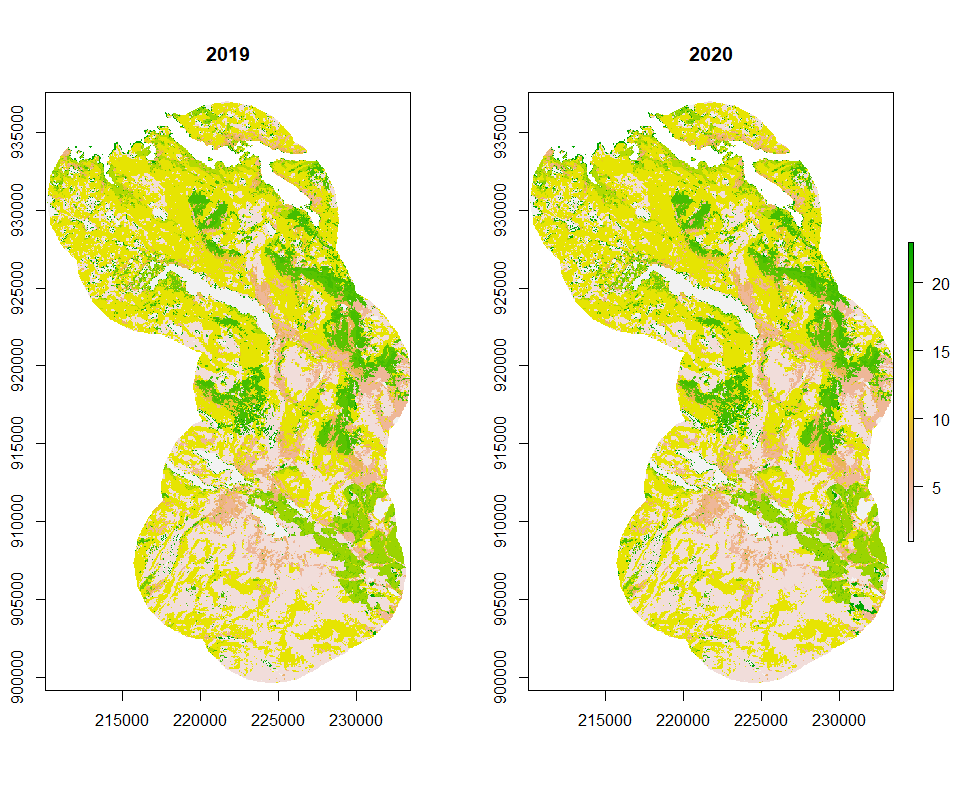


Figure 1. Landcover distribution in the Assynt study region given a 5 km buffer surrounding known habitat patches. These maps represent landcover in both 2019 and 2020 and are representative of the static nature of the habitat in the Assynt study region.

According to the metadata, the pixels have values from 1 to 8, coded as follows: 1: Agriculture related changes, 2: Afforestation, 3: Deforestation, 4: Forest growth, 5: Urban development, 6: Water gain, 7: Water loss, 8: Other changes. In the study area > 99.3 % of landscape did not experience change between 2019 and 2020 ; < 0.2 % of landscape experienced afforestation; < 0.1 % of landscape experienced deforestation; < 0.15 % of landscape forest growth < 0.3 % account for “other changes”, which could include satellite detection errors (Fig 2).


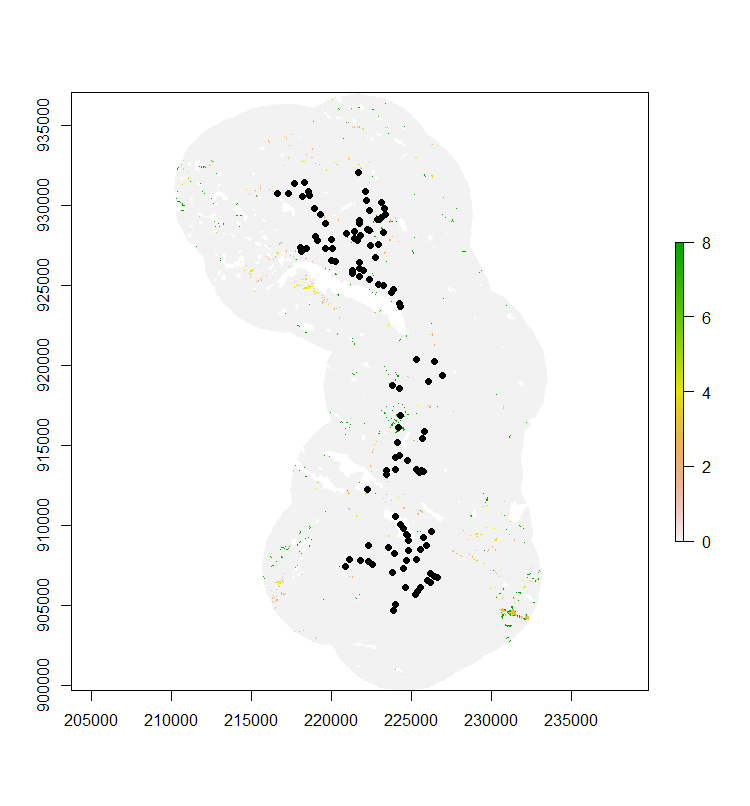


Figure 2. Total landcover change recorded in Assynt, Scotland accounts for less than 0.7% of landcover area in a 5k buffer region around known habitat patches (black points). Please refer to metadata provided by Space Intelligence (2021) to identify specific landcover change classifications which are broken in 8 distinct categories.

According to this satellite derived data, this is 9051 cells in the study area that experienced change. At a grain size of 20x20 meters, this equates to 3620400 m^2^ which at first instance may seem to be a large amount of landscape change, but this accounts for less than 0.7 % of the total habitats within a 5 km buffer surrounding our study area’s metapopulation patches.

As well, the vast majority of the change recorded has occurred well outside of likely dispersal distances or dispersal paths between our study patches (Figure 2). As well, much of this change is aggregated in the southwest corner of the buffered study area. Combined with the personal experience of the authors, the evidence suggests that landcover change is not occurring in a spatiotemporally relevant manner that may contribute to shifts in available habitat within the Assynt study area for the water vole (*Arvicola amphibius*).

NB - A single professional football pitch is ~ 7140 m^2^ (10m5X68m) and that means the total change in this area is about ~ 507 football pitches of change. Seems like a lot except that the 5km buffer is an area that contains over 122,781 pitches of space.

Works Cited

Space Intelligence, 2021. Habitat Maps of Scotland 2019 and 2020; <https://www.space-intelligence.com/2021/04/01/weve-just-published-the-first-ever-scotland-wide-high-resolution-habitat-maps-for-free/> [Accessed May 05, 2021]
